# “They can coach themselves essentially”: goalball coaches' perspectives on athlete-centered coaching with youth athletes with visual impairments

**DOI:** 10.3389/fspor.2026.1926809

**Published:** 2026-08-26

**Authors:** Wellington De Luna Vazquez, Luis Columna, Deborah Shapiro

**Affiliations:** 1 Department of Health Sciences and Human Performance, University of Tampa, Tampa, FL, United States; 2 Department of Kinesiology, University of Wisconsin-Madison, Madison, WI, United States; 3 Department of Kinesiology and Health, Georgia State University, Atlanta, GA, United States

**Keywords:** adapted sports, adolescents, coaching, paraSport, visually impaired

## Abstract

**Introduction:**

Coaching plays an important role in shaping the sport experiences of youth athletes with visual impairments (VI). Although athlete-centered coaching (ACC) has been proposed as an approach to support athlete involvement, development, and well-being, limited research has examined how ACC is understood and applied within youth disability sport.

**Methods:**

A descriptive qualitative design was used to explore goalball coaches' perspectives on ACC. Nine goalball coaches from the United States and Canada participated in semi-structured interviews conducted online or by phone. Interview data were transcribed verbatim and analyzed using reflexive thematic analysis.

**Results:**

Two themes were constructed: (1) “It Sounds Like What I'm Already Doing” and (2) “It's Harder Than Traditional Coaching.” Most coaches were unfamiliar with ACC as a formal concept, yet described coaching practices aligned with athlete-centered principles. Coaches also reported challenges applying ACC consistently, particularly when balancing athlete independence with structure, guidance, and competitive demands.

**Discussion:**

Findings suggest that goalball coaches value coaching approaches consistent with ACC, even without formal training or terminology related to athlete-centered coaching. However, coaches described uncertainty regarding the practical implementation of ACC within youth goalball environments. Greater education and coaching support may help facilitate more intentional application of ACC principles in disability sport settings.

## 1 Introduction

Participation in team sports has been consistently linked to higher levels of physical activity and improved physical and mental health outcomes for children and adolescents (1). For many children and youth, including those with visual impairments (VI), organized sport is a primary way to engage in regular physical activity. However, children and youth with VI are less likely to meet recommended physical activity guidelines (2, 3) and also participate in organized sports at lower rates than their peers without disabilities (4–6). This is concerning because organized sport is one of the few spaces where youth with VI can be physically active, build peer relationships, and receive structured support (7). Access to organized sport, as well as continued involvement over time, depends in part on the support available to youth with VI. This support includes access to programs, equipment, transportation, and coaching (8–10).

Coaching, in particular, plays an important role in how youth experience sport (11). For many youth with VI, coaches influence how activities are adapted, how instructions are communicated, and how athletes are included during training and competition (12, 13). These decisions affect who feels heard, how ability is understood, and how athletes learn. Over time, this influences whether youth with VI feel supported and capable, and whether they choose to remain involved (14). Because youth with VI often rely on coaches to explain instructions, modify activities, and support participation in sport environments designed primarily for sighted athletes, this influence is particularly strong (15). Athletes with disabilities, including those with VI, consistently report valuing coaches who understand disability and respond to individual needs (16). Meeting these expectations related to communication, adaptation, and attention to individual needs places significant demands on coaches working in disability sport, since many coaches receive limited preparation for working with athletes with disabilities, including VI (17, 18). In the absence of VI-specific training, coaches often draw on knowledge and strategies from able-bodied sport (19, 20). As a result, physical activity and sport experiences for youth with VI may be influenced by practices that do not always fully account for how visual impairment shapes participation and learning (3). One coaching framework proposed to better support athlete learning and development is athlete-centered coaching (ACC) (21).

In many sport settings, coaching has often emphasized coach control, directive instruction, and performance outcomes, with more limited involvement from athletes in decision-making (22). In response, ACC has gained attention as an alternative philosophy grounded in humanistic principles, emphasizing the development of the whole athlete by addressing both physical and psychological needs (23, 24). ACC is commonly described as an approach that encourages athlete involvement in learning through shared decision-making and active participation (25). Rather than directing all aspects of training, coaches using ACC invite athletes to contribute to decisions about goals, learning, and development.

The coaching philosophy of ACC has gained considerable attention as an alternative to traditional coach-directed approaches, its implementation is increasingly recognized as more complex than its underlying principles suggest (26). In youth sport, ACC has been associated with positive athlete outcomes, including greater confidence, independence, and identity development through increased involvement in learning and decision-making (27). These outcomes may be particularly relevant for youth with VI, who may have fewer opportunities to acquire certain skills through incidental learning. These practices could closely align with self-determination, which includes decision-making, problem-solving, goal setting, leadership, and self-advocacy. For example, the Expanded Core Curriculum identifies self-determination as an important area of development for youth with VI (28). Supportive physical activity and sport environments may provide meaningful opportunities to practice these skills (29, 30). Indeed, youth with VI have reported greater confidence in their self-determination within a sports-camp environment than at home or school (31). ACC may therefore provide a coaching framework through which youth with VI can practice self-determination through communication, shared decision-making, and increased ownership of their development. However, despite its growing prominence in coaching literature, ACC is not always clearly understood or consistently implemented in practice (32). Research suggests that coaches often experience difficulty translating athlete-centered principles into everyday coaching, particularly when balancing athlete involvement with time constraints (25), maintaining performance expectations in competitive environments (21), and determining when and how athletes should be involved in decision-making (33). More broadly, critical coaching scholars have questioned the notion that athlete-centered coaching represents a straightforward shift from coach-centered to athlete-centered practice (26). Rather, coaching has been described as an inherently relational, contextual, and ethically complex process in which coaches continually negotiate competing responsibilities (34). These challenges may be even more pronounced when coaching athletes with disabilities, where communication and participation often requires additional support or adaptations.

When ACC is applied without consideration of disability, coaches may unintentionally rely on assumptions about ability, independence, and success that do not reflect athletes' lived experiences (35). Consequently, opportunities for athlete involvement may continue to reflect coach priorities rather than athletes' perspectives. Coaching practices are always shaped by underlying assumptions about what constitutes effective coaching, assumptions that often remain unquestioned despite influencing how coaches define problems, make decisions, and exercise authority (34). In disability sport, these assumptions may be particularly consequential if they are informed primarily by able-bodied understandings of competence, independence, and success rather than by the experiences of athletes with visual impairments. Importantly, nearly all of these conceptual and practical discussions have been conducted in able-bodied sport. As a result, little is known about how coaches understand, interpret, and implement athlete-centered coaching in disability sport, including when working with athletes with visual impairments (36).

The limited evidence available in disability sport suggests that applying athlete-centered coaching may involve additional considerations beyond those described in mainstream sport. Many coaches receive limited preparation for working with athletes with disabilities, including those with visual impairments (17, 18). In the absence of disability-specific coach education, coaches often rely on coaching philosophies and practices developed in able-bodied sport (19, 20). However, coaching athletes with visual impairments frequently requires adapting communication strategies, instructional methods, and learning environments to meet athletes' individual needs (12, 13). Consequently, assumptions underlying athlete-centered coaching in mainstream sport may not transfer directly to disability sport. Despite these unique contextual demands, relatively little is known about how coaches conceptualize and implement athlete-centered coaching when working with athletes with visual impairments.

One disability sport that provides a particularly compelling context for examining these questions is goalball. As a Paralympic sport designed specifically for athletes with visual impairments, goalball presents coaching conditions that naturally amplify many of the central tensions surrounding athlete-centered coaching. During competition, coaches are prohibited from communicating with athletes while the ball is in play (37), requiring players to make tactical decisions, solve problems, and communicate with teammates independently. Athletes may also request time-outs and substitutions, and in many domestic competitions, teams may compete without a coach present (38). At the same time, coaches remain primarily responsible for athlete preparation, skill development, tactical instruction, and creating learning environments before competition (39). This coaching context is further complicated by the fact that many goalball coaches are sighted and may not fully understand the lived experiences or the full spectrum of challenges associated with visual impairment (40). As a result, coaches must continually interpret athletes' needs while balancing guidance with opportunities for athlete independence. Rather than simply representing another disability sport, goalball offers a context in which the practical tensions between athlete autonomy and coach guidance are inherent to the sport itself, making it an especially valuable setting for examining how coaches conceptualize and implement athlete-centered coaching.

Unlike many disability sports that have parallel able-bodied versions, goalball is unique. As a result, coaches cannot simply draw upon an established sport-specific coaching tradition from mainstream sport. Instead, many youth goalball coaches enter coaching through volunteer pathways and receive relatively limited formal coach education, often relying on broader coaching principles while adapting them to the unique demands of goalball. These characteristics raise important questions about how coaches understand and implement athlete-centered coaching in practice. Rather than assuming that principles developed in able-bodied sport transfer directly to disability sport, there is a need to examine how coaches themselves conceptualize athlete-centered coaching, how these understandings develop through lived coaching experiences, and how they navigate the practical challenges of balancing athlete empowerment with coach guidance. Collectively, the existing literature suggests that athlete-centered coaching is not simply the adoption of a coaching philosophy, but a context-dependent process requiring coaches to continually negotiate athlete autonomy, learning, performance, and responsibility. Although these conceptual and practical tensions have been widely discussed in able-bodied sport, it remains unclear whether they are experienced similarly when coaching youth athletes with visual impairments. Examining goalball coaches therefore offers an opportunity not only to address an important gap in disability sport research but also to determine whether current conceptual understandings of athlete-centered coaching are transferable to a context in which athlete independence, communication, and coach involvement are fundamentally different. Such an examination has the potential to extend existing athlete-centered coaching literature by exploring whether these theoretical tensions are reflected, modified, or challenged within youth goalball. Therefore, the purpose of this study was to examine goalball coaches' perspectives on ACC when working with youth athletes with visual impairments. The following research questions guided this study:

1. How do goalball coaches conceptualize athlete-centered coaching (ACC) when working with youth athletes with VI?
2. What challenges, if any, do goalball coaches describe when using ACC with youth athletes with visual impairments?

## 2 Materials and methods

### 2.1 Study design

This study was informed by an interpretivist research paradigm, which assumes that knowledge is co-constructed through social interaction and lived experience (41). Within this perspective, a descriptive qualitative methodology was used to explore how goalball coaches understand and apply ACC when working with youth athletes with VI (42). This approach allowed for in-depth descriptions of coaches' perspectives and experiences in youth goalball. To date, Research on coaching in youth goalball remains limited. Accordingly, this study served as an exploratory step toward better understanding how coaching philosophies are interpreted and enacted by goalball coaches in disability sport settings.

### 2.2 Participants

Ethical approval was obtained from the Georgia State University Institutional Review Board before participant recruitment. A purposive sample of goalball coaches were recruited through blind sport organizations, local goalball clubs, social media groups within the goalball community. Goalball coaches were required to (a) be 18 years or older and (b) have at least one year of experience coaching youth goalball athletes at local, regional, national, or international competitive events.

The final sample consisted of nine goalball coaches (three women and six men) from the United States and Canada. Their coaching experience ranged from 1 to 25 years (*M* = 6.44, *SD* = 7.84), and one coach self-identified as blind or having low vision. Participants represented diverse racial and ethnic backgrounds and had coached across local, national, and international settings. Additional coach-level characteristics, including formal preparation, goalball-specific training, coaching settings, and experience with individuals with visual impairments, are presented in Supplementary Table S1. To protect participants' anonymity within the small and interconnected goalball community, potentially identifying characteristics are reported using broad categories.

Coaches were first contacted via email and provided with a recruitment flyer and study information. Those who expressed interest were then sent an online consent form. Informed consent was obtained prior to participation. Participants received a gift card as compensation for their time. Given the small and interconnected goalball coaching community, some demographic details (e.g., education level and race) were withheld to protect participant anonymity.

### 2.3 Data collection

Data were collected from two sources: (1) a demographic questionnaire and (2) semi-structured interviews. The demographic questionnaire gathered information on participants' age, gender, years of coaching experience, education, and formal coach training.

Following the questionnaire, participants completed one-on-one semi-structured interviews conducted online via Zoom (*n* = 6) or by phone (*n* = 3), based on participant preference. Interviews lasted approximately 60–90 min and were audio-recorded.

The interview guide was developed based on the ACC literature and the study's research questions. To ensure content validity, the protocol was reviewed by a panel of four experts in kinesiology, disability sport, and sport coaching. Feedback from the panel, including minor revisions such as reordering questions, was incorporated prior to data collection. Example questions included: (a) Had you heard the term athlete-centered coaching before participating in this study? If so, how would you describe it in your own words? If not, how would you describe the way you typically coach youth goalball athletes?; (b) Can you walk me through what a typical practice or competition looks like for you when coaching youth goalball?; (c) What situations do you find most challenging when coaching youth goalball athletes, and how do you usually respond to those challenges?; and (d) How do you decide whether your coaching is meeting the needs of your athletes? During and after the interviews, the lead author kept reflective notes to capture key points and additional observations (43, 44).

### 2.4 Data analysis

All nine interviews were transcribed verbatim prior to analysis. The first author conducted the data analysis using reflexive thematic analysis (45, 46). Analysis began with careful reading and re-reading of the transcripts to become familiar with the data. Initial codes were generated inductively and grouped into broader categories. These categories informed the development of preliminary themes, which were refined through iterative review (45, 47). Reflective notes were kept throughout data analysis to capture observations that stood out and to identify areas for follow-up or further consideration. reflections. The first author shared the identified themes and supporting quotes with the research team for discussion, which led to the clarification and refinement of the theme descriptions. The final thematic structure was then agreed upon by the authorship team prior to inclusion in the study findings.

### 2.5 Trustworthiness and positionality statement

Several strategies were employed to enhance the trustworthiness of this qualitative study, drawing on the criteria of credibility, dependability, and confirmability (48). Credibility was supported through peer debriefing. The lead author discussed the analysis with colleagues who were not directly involved in the study. These conversations provided a space to reflect on interpretations and clarify the fit between the data and the research questions. Dependability was addressed through a peer checking. After initial coding, a second researcher reviewed the coded transcripts and proposed potential categories, that later became themes. The researchers then met to discuss areas of agreement and difference and to refine the thematic structure. Confirmability was supported through reflexive journaling. The lead author kept notes throughout data collection and analysis to remain attentive to how personal experiences and perspectives may have influenced the research. Participant quotes were used to illustrate themes and reflect participants' perspectives.

The lead author is an Assistant Professor and was also a goalball coach at the time of the study. His coaching background likely shaped interactions with participants and supported open conversations, while also carrying the potential to influence how participants described their experiences. Throughout the research process, the lead author used reflective notes and engaged with the research team in ongoing discussions to consider how their backgrounds and roles may have influenced data collection and interpretation (43, 49).

## 3 Results

Two major themes were constructed from the data: (1) “It Sounds Like What I'm Already Doing” and (2) “It's Harder Than Traditional Coaching.” Each theme includes related subthemes that reflect coaches' experiences and perspectives when working with youth goalball athletes with visual impairments. Pseudonyms were used for all participants.

### 3.1 “It sounds like what I'm already doing”

When coaches were asked about ACC, many said they were not familiar with the term. A few recalled hearing about it through coach education or certification. Coaches often described their coaching in ways that aligned with ACC principles. For many, ACC was not something they thought of as a formal framework but something that felt familiar in their day-to-day work with youth goalball athletes. As they talked about their coaching, coaches emphasized ideas such as involving athletes in decisions, being flexible in practice, and paying attention to athletes as individuals rather than focusing only on performance. This theme is supported by three subthemes: (1) Unfamiliar with the term, not the ideas, (2) More than just winning, and (3) Giving athletes a say.

#### 3.1.1 Unfamiliar with the term, not the ideas

Most coaches said they were not familiar with ACC as a formal concept before taking part in the study. Several spoke about hearing the term for the first time. Joseph shared, “I don't think I had ever heard of athlete-centered coaching before reading the [consent form] earlier today.” Nia echoed this lack of familiarity. She said, “No, I haven't heard that specifically, but please tell me about it.” Some coaches were brief and direct when describing their unfamiliarity with the term. Harold said that he “had not heard the term before”. Zavier also described limited familiarity. He explained, “I have heard of it, but no, I know I will not know the definition of that.”

A smaller number of coaches recalled some prior exposure to ACC, most often through coach education or certification. Andre noted that the concept sounded familiar. He said, “It sounds familiar. I don't have to do much about it, but when I was trying to get my certificate, we were briefed about it.” Jenna also described having some awareness of the concept but without having explored it in depth. She said, “I am not completely familiar with it, but I know there's been stuff out there, but I haven't fully researched it.” Kevin noted that while the term itself was unfamiliar, the ideas behind it felt recognizable to him. He said, “You might be able to tell me the philosophy behind it. It might seem familiar to you, but the actual phrase, athlete-centered coaching, is not something I'm familiar with.” Joseph also reflected that while parts of ACC felt familiar, he did not see all aspects of his coaching as fully athlete-centered. He noted, “I probably would as far as drills and our time together as a team. I tend to be more controlling over that than athlete-centered coaching seems like it would be.” Despite this limited familiarity with the term, coaches often went on to describe their coaching in ways that reflected ACC principles. While the language of ACC was not widely used, many of its ideas appeared to be present in how coaches worked with youth goalball athletes.

#### 3.1.2 More than just winning

Coaches described ACC as extending beyond performance outcomes and winning. For many, success in youth goalball was closely tied to athlete growth, confidence, and learning, rather than game results alone. ACC was often described as a way of supporting athletes as people, not just as competitors. Nia spoke about ACC as a way of supporting athletes as people, not just as competitors. She explained, “[Athlete-centered coaching] is better for the sport, it's better for the team it's better for the outcome, but it's better for the whole person.” She also stressed that ACC needed to be flexible and responsive, noting that it was “not one size fits all.” Nia also cautioned against treating ACC as a formula. She added,

“If you're acting there's only one way to do athlete-centered coaching or these are the ingredients that always, in the same amounts, that always result in athlete-centered coaching, I think you're not approaching it thoughtfully, and therefore you're not actually doing athlete-centered coaching.”

Several coaches linked ACC to helping athletes develop confidence and independence over time. Theo spoke about this focus as central to how he approaches coaching, describing his work as aimed at supporting athletes beyond performance alone. He noted, “It's basically almost what I do. It's to improve the athletes and to improve the overall well-being.” Nia also emphasized empowerment as a key part of coaching, describing it as something that extended beyond the sport itself. She asserted,

“I love the idea of empowerment as an important function of a coach. Because you'll hear people in sports a lot of the time talk about this is goalball, but it's also about life. Empowering people to make choices on the court means we're empowering people to make choices off the court, empowering people to see themselves as being really competent and being able to do much more than maybe they thought that they could.”

Coaches described a tension between supporting athlete decision-making and pursuing competitive results. Joseph recalled a tournament where giving athletes more control meant accepting less favorable outcomes in the short term. He described,

“Our team finished 0–4… It could have been slightly better in a couple of circumstances if I had told them what to do, but they would not have had as much fun, they wouldn't have been as empowered. The trade-off is definitely worth it.”

For Joseph, prioritizing athlete involvement and confidence mattered more than the final score. Other coaches, however, emphasized that winning itself could be an important source of empowerment. Andre pointed to competitive success as meaningful for youth athletes. He stated,

“Nothing empowers a teenager more than the feeling of winning. Make sure that you believe in them as much as they do themselves, or even when they don't believe in themselves, you have to show them that you believe in them and they can win.”

Andre also described coaching and sport participation as extending beyond competition, noting that goalball provided a sense of purpose and belonging. He shared,

“Goalball is like an escape. It's an escape for me out of the real world. It gives me a sense of inspiration… It empowers me as much as I feel I'm empowering the kids. It gives me a sense of belonging to my society wherever I am.”

#### 3.1.3 Giving athletes a say

Coaches described fostering athlete ownership as a central component of how they understood and practiced ACC. Rather than simply encouraging athletes to express their opinions, coaches emphasized preparing youth athletes to think independently, make informed decisions, reflect on their experiences, and gradually assume greater responsibility for their own development. Theo described ACC as placing athletes at the center of decision-making, explaining that it required athletes to be active participants rather than passive recipients of instruction. He noted,

“I feel like athlete-centered coaching is a philosophy that places the athlete in making decisions and all the activities, based on the athletes' individual needs and development. They have to think and take ownership of those decisions, and they also have to be intentional about their development.”

Joseph described involving athletes in decisions about their roles and development through ongoing conversations, while also recognizing when he needed to step back as a coach. He explained,

“I don't necessarily just tell them, ‘play whatever position you want,’ but I have a lot of conversations with them, asking them, ‘Which position would you like to play? And which players would you want to play with?’ When an athlete tells me what they want to do, it gives them a great sense of being included in the process and not just being like a soldier. I probably would, as far as drills and our time together as a team, tend to be more controlling than athlete-centered coaching seems like it would be.”

Other coaches described athlete involvement as something that happened naturally within goalball. Kevin explained that he often adjusted practices based on athlete input, even when it meant changing his original plan. He said,

“Sometimes they'll ask me, ‘Can we work on rolling today?’ or ‘Can we work on penalty shots today?’ and maybe you run with a practice that wasn't what I think we're working on today, but I'm fine being flexible and allowing them to do that. Goalball lends itself more than other sports. They can coach themselves essentially. They're basically the players running the show themselves. The players on the court know what to do and how to do it… they don't need a coach to tell them they got four seconds left to roll the ball. They know it, they know what's going on.”

Kevin also noted that this flexibility was closely tied to enjoyment. He added, “I kind of do some of that just naturally, not doing it on purpose. I want them to have fun.” Jenna described giving athletes a say as offering choice within clear boundaries, rather than removing structure altogether. She emphasized that athletes responded more positively when they felt some control over their learning, especially when practices reflected their needs. She asserted,

“They respect you more when you do give them that little bit of athlete-centered focus. As long as they think maybe they're in control, you're giving them choices—but you're giving them choices that you are okay with. Athletes can pick their own drills. They may know what they want to work on this week. I'm a coach, I'm not playing the sport. They know best what they need to work on. If I'm picking a drill that doesn't even focus on that, then that's not going to help them, and they're just going to be frustrated at the practice.”

### 3.2 “it's harder than traditional coaching”

While many coaches described athlete-centered coaching (ACC) as consistent with how they wanted to work with youth goalball athletes, they also talked about how demanding it could be in practice. Coaches explained that using ACC required them to constantly make decisions, adjust their plans, and think carefully about when to step in and when to step back. This was especially challenging when working with youth athletes who differed in experience, confidence, and readiness to make decisions on their own. Coaches also described the mental demands of staying flexible, responding to athletes' needs in the moment, and figuring things out without much formal guidance or training. This theme is supported by three subthemes: (1), Knowing when to step in (2) It takes a lot more out of you, and (3) Learning as you go.

#### 3.2.1 Knowing when to step in

Coaches described uncertainty about how much direction to provide when working with youth goalball athletes. Although athlete involvement was viewed as important, coaches noted that not all athletes were ready to make decisions independently, particularly when they lacked experience or confidence. Kevin questioned whether athletes always had the game knowledge needed to guide their own play. He explained,

“The challenge of it is, are they knowledgeable of the game? I've seen teams out there where you have three individuals that are not playing as a team. They're all out, they're all ball hogs. I wouldn't want them using an athlete-centered approach because I don't think they're gonna play good.”

Concerns were also raised about athletes' willingness or ability to communicate their needs. Theo spoke about situations in which athletes struggled to express themselves. He noted, “Some of these players are not really vocal. Some of them don't really voice things they don't like. There's a lot of psychological things. Most of the challenges we have are most psychological.” Stella described the limits of athlete self-direction and emphasized that ACC required ongoing judgment based on the athlete and the coach–athlete relationship. She commented,

“If it couldn't be self-directed for the athlete, you would support them to do that, but it may not be athlete-centered. It's not a new term. I think it's also case by case because every athlete comes with a different set of athletic sense, confidence, sportsmanship. I think there's a combination of athlete-centered delivery and coaching, but it's also, there's a trust factor there.”

Jenna also emphasized that youth athletes often continue to look to coaches for direction and reassurance. She explained, “Youth are still learning and want your guidance and are reaching out to you and want you there.”

#### 3.2.2 It takes a lot more out of you

Many coaches described ACC as more demanding than more traditional ways of coaching. Compared to traditional coaching, ACC required sustained attention, flexibility, and responsiveness to athletes' needs. Nia spoke about the effort involved in trying to coach this way, describing it as something she continued to work toward rather than something she felt she had mastered. She shared,

“It's harder than just traditional coaching can be. I'm not saying I get it right all the time, but it's what I aspire to all the time… If you really, really want to do athlete-centered coaching, it's a lot of mental homework.”

Kevin also spoke about the demands of responding to athletes' moods, energy levels, and attitudes on a day-to-day basis. He described how quickly a practice plan could change based on what athletes brought with them that day. He explained,

“There are gonna be days where they have good attitudes, days with bad attitudes, and you just got to roll with it. You might have the perfect practice planned, and within three minutes of it, you realize you have to toss out the whole plan.”

#### 3.2.3 Learning as you go

Coaches also described challenges related to limited preparation and support when attempting to use ACC. Several felt that limited access to formal training made it difficult to apply athlete-centered ideas in a consistent way. Nia spoke about how challenging this could be for coaches who were starting programs or working with minimal guidance. She explained,

“It's tough for coaches who are maybe by themselves starting a program or have very limited training. We see it right, especially in youth competitions, where a lot of teams clearly lack some understanding of the basics of the sport. I don't know how you can really do athlete-centered coaching with regard to competence, particularly if you don't have that background yourself. I think coach education can be a very big stumbling block.”

Joseph described learning through experience, particularly when dealing with expectations from outside the team. He shared that parents sometimes questioned his coaching decisions. He commented, “I have gotten some pushback from family members, like parents, who think that something should be done in a certain way or think we should have done something differently.”

In summary, participants described ACC as something that often felt familiar in their day-to-day coaching, even when the term itself was new. Coaches valued involving athletes in decisions, supporting autonomy, and emphasizing development beyond winning. At the same time, they spoke about the challenges that came with this way of coaching, including uncertainty about when to step in, the mental effort required to stay flexible, and limited access to formal preparation or guidance.

## 4 Discussion

The purpose of this study was to examine goalball coaches' perspectives on athlete-centered coaching (ACC) when working with youth athletes with visual impairments. Although most coaches were unfamiliar with ACC as a formal concept, they consistently described coaching practices that aligned with its core principles. The findings suggest that coaches developed these practices primarily through experience rather than formal coach education and viewed implementing ACC as a continual process of adapting guidance to athletes' individual needs and readiness. Within the unique context of goalball, athlete-centered coaching extended beyond promoting athlete voice to preparing athletes to think and perform independently when coach support was unavailable during competition. The following discussion is organized around the research questions that guided the study.

### 4.1 RQ 1: how do goalball coaches conceptualize athlete-centered coaching (ACC) when working with youth athletes with VI?

Rather than defining ACC in formal terms, coaches described it through how they related to athletes and how they made everyday coaching decisions. They emphasized listening, responding to individual needs, and helping athletes grow over time. Success was framed in terms of confidence, independence, and enjoyment, rather than outcomes alone. These priorities align with existing work on athlete-centered coaching, which emphasizes autonomy, shared decision-making, and attention to the whole athlete (50, 51).

How goalball coaches came to understand ACC was closely tied to how they learned to coach, which is consistent with previous research (26). Coaches described developing these values primarily through experience, often by paying attention to athletes' responses and adjusting their coaching over time, rather than through formal coaching training. This is common in disability sport, where coaches frequently enter through non-traditional pathways and have limited access to formal coach education (52). In this study, understanding of ACC developed through ongoing interaction with youth athletes with VI rather than through shared terminology or structured instruction (53). Because coaches described learning ACC primarily by working with athletes, experience alone may be insufficient to support a more intentional understanding and use of ACC in youth goalball.

What coaches described also helps explain why familiarity with ACC was limited. Many goalball programs rely on volunteer coaches who are motivated by passion, proximity, or personal experience with disability rather than formal preparation (54). As a result, many coaches learned to coach on the job and developed their coaching philosophy through experience rather than structured training. Previous research showed that coaches in both able-bodied and disability sport often struggled to clearly define or consistently apply ACC in practice. This occurred even when coaches valued principles such as athlete involvement, shared decision-making, and development beyond winning (32, 55–57). For youth goalball, this suggests a need for clearer guidance and support so that ACC does not depend solely on individual experience.

### 4.2 RQ 2: what challenges, if any, do goalball coaches describe when using athlete-centered coaching (ACC) with youth athletes with visual impairments?

Many coaches found implementing ACC challenging. These concerns align with prior research suggesting that ACC can be more mentally demanding than traditional coaching, as it often requires greater preparation, flexibility, and emotional awareness (58). In this study, coaches explained that these challenges emerged in everyday coaching situations, where they had to decide when to step in, when to step back, and how much guidance to provide to different athletes. In goalball, these decisions were further influenced by athletes' sensory needs and varying levels of experience. Coaches were not struggling with whether ACC was important, but with how to apply it consistently while supporting athlete independence and team success. These findings suggest that coaches need training for making these day-to-day decisions, instead of relying solely on trial and error.

In addition to these practical demands, coaches described uncertainty about how to define and evaluate success when using ACC. Several coaches emphasized empowerment as an important marker of success. They placed greater value on athletes' confidence, independence, and decision-making than on winning games alone. While competition still mattered, success was often described as youth athletes taking greater ownership of their learning and development over time. These outcomes align with the core aims of athlete-centered coaching, which emphasize developing the whole person rather than focusing solely on performance (27, 59). At the same time, the lack of clarity around what counts as success suggests that coaches may benefit from more specific training on how to recognize and support these outcomes in practice, particularly in competitive youth sport settings. Existing ACC literature has largely focused on how coaches provide athletes with greater autonomy while remaining the central decision-makers throughout training and competition. In contrast, goalball structurally redistributes many coaching functions to athletes during competition. Because coaches are prohibited from communicating while the ball is in play, athletes must independently solve tactical problems, communicate with teammates, regulate performance, and adapt to changing game situations in real time. As one coach described, “they can coach themselves essentially.” Rather than representing the absence of coaching, this finding suggests that coaching in goalball becomes distributed across coaches and athletes. Coaches prepare athletes before competition, whereas athletes assume greater responsibility for tactical decision-making during play. Consequently, athlete-centered coaching in goalball may involve preparing athletes to coach themselves rather than simply providing greater opportunities for athlete choice. Preparing goalball athletes to “coach themselves” may also have implications beyond sport-specific performance. The communication, decision-making, problem-solving, leadership, and self-advocacy described by coaches overlap with self-determination and social-interaction skills emphasized within the ECC (8). Coaching goalball through an ACC lens may create a naturally occurring environment in which youth with VI practice communicating their needs, negotiating team roles, responding to feedback, and making decisions under pressure. These skills may extend beyond training and competition, potentially preparing athletes to navigate educational, workplace, and other social environments. Previous research has similarly identified sport environments as potential settings for supporting self-determination among youth with VI (31).

Some coaches described success not only in terms of athlete outcomes, but also in feeling that their coaching had purpose and meaning. Supporting youth athletes and seeing them stay engaged in goalball reminded these coaches why they chose to coach in the first place. For them, empowerment was connected to the relationships they built with athletes over time, rather than to performance outcomes alone. How coaches defined success was also influenced by their backgrounds, particularly those that emphasized participation and inclusion. As a result, coaches did not always approach ACC with the same goals or expectations, even when they shared similar values. These differences suggest that the field would benefit from a shared understanding of how empowerment, development, and competition fit within ACC success in youth goalball. The findings of this study do not suggest that ACC should replace attention to performance. Instead, coaches described adjusting how they coached based on athletes' readiness, maturity, and the competitive situation. In some cases, this meant providing more structure, while in others it meant stepping back to preserve athlete voice and involvement. The challenge for coaches was not choosing between empowerment and performance, but figuring out how to support both at the same time. Many coaches expressed uncertainty about how to do this in practice. These findings highlight the importance of providing practical support that helps coaches decide when to step in, when to step back, and how to apply ACC values during everyday coaching situations, especially during tension to avoid discouragement or burnout (60).

### 4.3 Limitations of the study and future research

To our knowledge, this study is one of the first to examine how goalball coaches conceptualize and experience ACC when working with youth athletes with VI. Despite this contribution, several limitations should be considered when interpreting the findings. First, the primary researcher's role as a goalball coach may have influenced both data collection and analysis. Although steps were taken to minimize bias, the researcher's dual role as a goalball coach and investigator may have influenced how participants shared their experiences and how the data were interpreted. Second, this study focused intentionally on coaches' perspectives and did not include the experiences of athletes, parents, or others involved in youth goalball. Focusing on coaches allowed for an in-depth examination of coaching beliefs. However, not including athletes or parents limits understanding of how ACC is experienced and supported in youth goalball.

Future research is needed to further examine athlete-centered coaching in youth disability sport. Studies could explore how ACC is understood and used across different blind sports to identify shared challenges and sport-specific differences. Research is also needed to examine how coach education, lived experiences as Para athletes (if any), and educational background influences the use of ACC in practice. For example, future studies could investigate whether infusions of instructional approaches such as the Expanded Core Curriculum, Paralympic Sport Day and Sport Education Model support athlete involvement, decision-making, and confidence among youth with VI (61). Longitudinal studies that include both coach and athlete perspectives may also help clarify how ACC develops self-determination over time and how it could blend effectively with other models (e.g., Long-Term Athlete Development or Expanded Core Corriculum).

## 5 Conclusion

The purpose of this study was to examine goalball coaches' perspectives on athlete-centered coaching (ACC) when working with youth athletes with visual impairments. Although most coaches were unfamiliar with ACC as a formal concept, they consistently described coaching practices that aligned with athlete-centered principles. Rather than understanding ACC through formal frameworks, coaches conceptualized it through everyday relationships, ongoing interactions, and context-specific coaching decisions. At the same time, they highlighted the complexity of implementing ACC in practice, particularly when determining how much guidance to provide while fostering athlete independence and preparing athletes for competitive performance. Coaches also differed in how they defined success, placing varying emphasis on athlete development, empowerment, and performance outcomes. Collectively, these findings suggest that athlete-centered coaching in youth goalball is less about adopting a particular coaching philosophy than about continually adapting coaching practices to the developmental needs of athletes and the unique demands of the sport. Supporting coaches in developing this context-sensitive judgment may strengthen the intentional application of athlete-centered coaching in youth disability sport.

## Data Availability

The datasets presented in this study can be found in online repositories. The names of the repository/repositories and accession number(s) can be found below: https://doi.org/10.17605/OSF.IO/5CQ3M.
