# Supplementary material for: “They can coach themselves essentially”: goalball coaches' perspectives on athlete-centered coaching with youth athletes with visual impairments
Source: Front Sports Act Living. 2026 Aug 26;8:1926809. doi: 10.3389/fspor.2026.1926809 (PMC13556155; doi:10.3389/fspor.2026.1926809)
Supplement: Supplementary file 1 [file Table1.docx]

Supplementary Material

| Participant | Years of experience | Education Level | Certified Coach in Disability Sports | Formal Training in Coaching | Highest Level of Tournament Participation as a Coach | Experience coaching able-bodied athletes in mainstream sports |
| --- | --- | --- | --- | --- | --- | --- |
| Stella | 2 | Bachelor's degree | Yes | Yes, specific to coaching goalball. | State/Regional Tournaments | Yes |
| Kevin | 8 | Master's degree | No | No | National Championships | Yes |
| Zavier | 4 | Bachelor's degree | No | Yes, specific to coaching goalball. | National Championships | No |
| Jenna | 12 | Bachelor's degree | Yes | Yes, but not specific to coaching goalball. | International Competitions * | No |
| Nia | 2 | Doctorate | No | Yes, specific to coaching goalball. | National Championships | Yes |
| Joseph | 2 | Master's degree | No | Yes, but not specific to coaching goalball. | National Championships | No |
| Harold | 25 | Bachelor's degree | No | No | International Competitions * | Yes |
| Anton | 2 | Bachelor's degree | Yes | Yes, specific to coaching goalball. | State/Regional Tournaments | No |
| Theo | 1 | Bachelor's degree | No | Yes, specific to coaching goalball. | Local/Community Club Tournaments | Yes |

# 1 Table – Participants’ Demographics

*Notes: *(e.g. Youth Worlds, Youth Para Panamerican Games)*
